# Effective treatment of locally advanced periocular basal cell carcinoma with oral hedgehog pathway inhibitor?

**DOI:** 10.1007/s00417-020-04779-5

**Published:** 2020-06-09

**Authors:** Xiaoyi Hou, Alexander C. Rokohl, Monika Ortmann, Ludwig M. Heindl

**Affiliations:** 1 Department of Ophthalmology, Faculty of Medicine and University Hospital Cologne, University of Cologne, Cologne, Germany; 2 Department of Pathology, Faculty of Medicine and University Hospital Cologne, University of Cologne, Cologne, Germany; 3 Center for Integrated Oncology (CIO) Aachen-Bonn-Cologne-Duesseldorf, Cologne, Germany

**Keywords:** Basal cell carcinoma, Eyelid carcinoma, Eyelid, Hedgehog pathway inhibitor, Sonidegib

Dear Editor,

Basal cell carcinoma (BCC) is the most common malignant tumor in the periocular region [1, 2]. The vast majority of periocular BCCs can be effectively treated by complete histopathology-controlled excision, the gold standard for BCC treatment [3]. However, in some cases, alternative approaches are required due to advanced stage of the BCC, reduced general condition of the patient prohibiting general anesthesia, unreasonable cosmetic changes after surgery, or multiple BCC lesions such as in Gorlin-Goltz-syndrome [4]. These alternative approaches include among others systemic therapy with immune checkpoint inhibitors such as sonidegib [4, 5].

Sonidegib (Odomzo®), an oral hedgehog pathway inhibitor (HPI), is indicated for the treatment of adults with locally advanced BCC (laBCC) that are not candidates for surgery or radiation therapy or adults with recurrent laBCC following surgery or radiation therapy [6, 7]. Until today, there is no report of sonidegib for treating periocular laBCC, and evidence regarding optimal management of this condition is still limited. Hence, we demonstrate a case of a laBCC on the lower eyelid margin which was successfully treated with sonidegib.

**Fig. 1:**
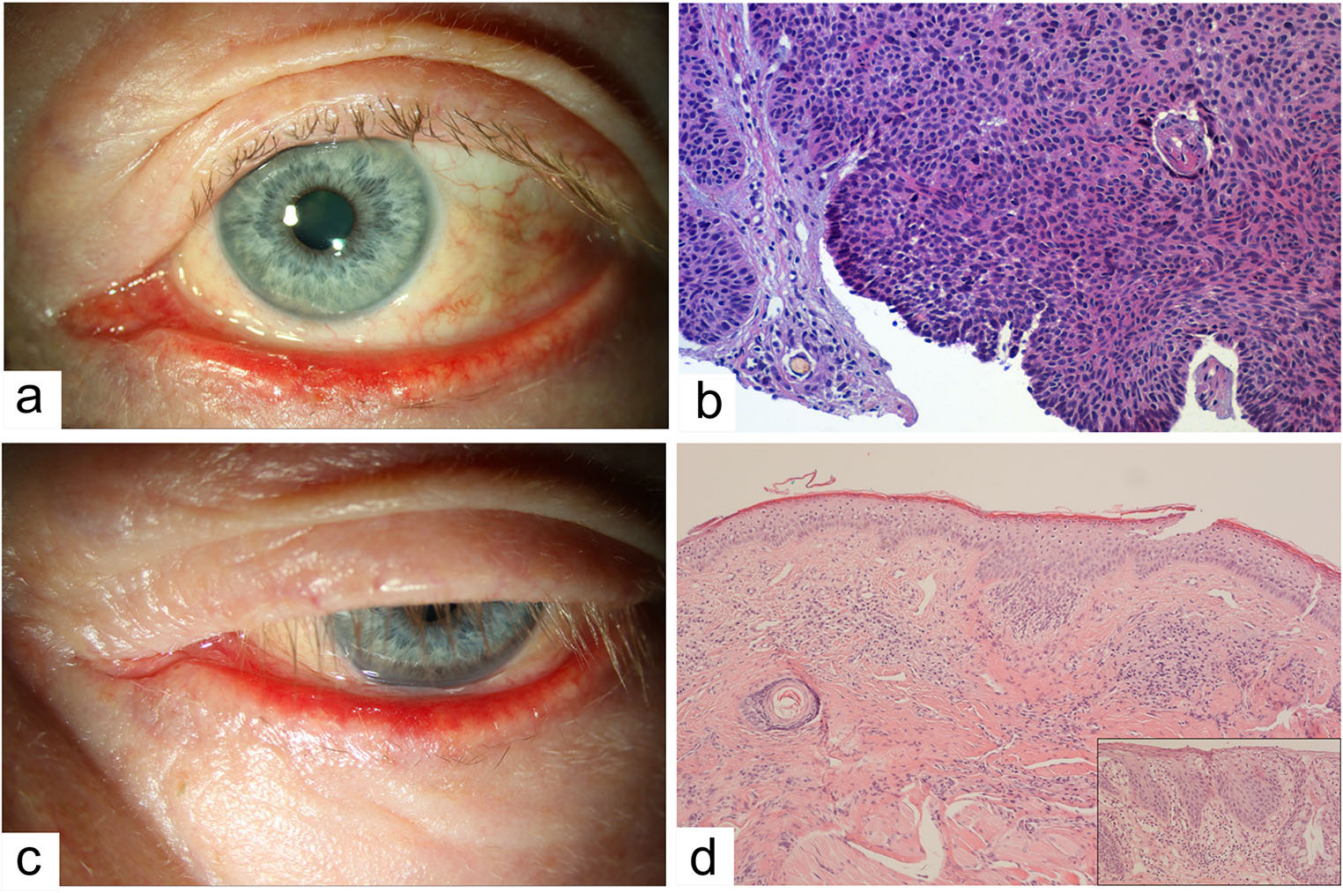
Lower eyelid basal cell carcinoma (BCC) treated with sonidegib. **a** A 73-year-old man with a 20-year history of multiple BCCs at his back, chest, nose, and ear presented with marked blepharitis and ulcered lesion in the center of the lower eyelid margin with clinical suspicion of a morphea-type BCC. **b** Hematoxylin-eosin (HE) staining of the biopsy shows basaloid nests as well as typical gap formation between tumor cell nests and the surrounding stroma (arrow) (HE: × 10), compatible with BCC. **c** Following treatment with sonidegib for 6 months, there was marked persisting blepharitis and lower eyelid ectropion, suspicious for BCC recurrence. **d** HE staining of the second full-thickness biopsy shows tiny foreign body granulomas and neutrophil adjacent to the deep epidermis (HE: × 10). Insert demonstrates the eyelid margin was edematically irritated without stronger layering disorder or cytological atypia, presenting with hypokeratosis (HE: × 20), ruling out BCC recurrence

A 73-year-old man with a 20-year history of multiple BCCs at his back, chest, nose, and ear presented with a morphea-like BCC of the left lower eyelid along with an ectropion (Fig. 1a). Local biopsy revealed histopathologically confirmed BCC (Fig. 1b). The patient refused any further surgical or radiotherapeutic interventions and preferred systemic treatment. After oral treatment with sonidegib (capsule 200 mg; Odomzo ®) once a day for 6 months, the lesions on the eyelid margin showed no significant clinical remission, and the lower eyelid ectropion persisted (Fig. 1c). Due to the suspicion of a persistent malignant process and in order to treat the ectropion, a full-thickness biopsy of the lower eyelid was performed. The histopathology revealed no manifestation of a BCC anymore but only nonspecific inflammation and slight parakeratosis (Fig. 1d).

Ninety percent of BCCs have a pathologic activation of the sonic hedgehog pathway; vismodegib is the first HPI been applicated in the treatment of periocular laBCC [8]. However, during treatment with vismodegib, new squamous cell carcinomas (SCC) of the skin were seen [8], and it is currently still unclear whether these SCCs were a side effect or a coincidence of BCC. Sonidegib (200 mg), the second hedgehog inhibitor approved, was successfully applied for use in patients with laBCC [6] based on the meaningful, durable tumor responses observed in the BCC outcomes with the BOLT study [9]. Nevertheless, there is no study investigating the effectiveness of sonidegib for treating periocular laBCC until now.

Our patient was the first case of a successful treatment of oral HPI for periocular laBCC. In our case, histopathology-confirmed complete remission was achieved under therapy with 200 mg sonidegib with only slight side effects, including muscle spasms and vomiting. A second malignancy did not occur after 6 months treatment with sonidegib. Hence, our report suggests that sonidegib seems to be an effective treatment option for periocular laBCCs without the side effect of secondary malignancies. However, long-term efficacy and complications have to be investigated in further studies with a larger population.
